# Clinical characteristics and prognostic analysis of acute necrotizing encephalopathy of childhood: a retrospective study at a single center in China over 3 years

**DOI:** 10.3389/fneur.2023.1308044

**Published:** 2023-12-20

**Authors:** Yu Fang, Qiqi Gao, Wenwen Jin, Jianshun Li, Hao Yuan, Zhenlang Lin, Guoquan Pan, Wei Lin

**Affiliations:** ^1^Department of Pediatrics, The Second School of Medicine, The Second Affiliated Hospital and Yuying Children’s Hospital of Wenzhou Medical University, Wenzhou, Zhejiang, China; ^2^Key Laboratory of Perinatal Medicine of Wenzhou, The Second Affiliated Hospital and Yuying Children’s Hospital of Wenzhou Medical University, Wenzhou, Zhejiang, China; ^3^Key Laboratory of Structural Malformations in Children of Zhejiang Province, The Second Affiliated Hospital and Yuying Children’s Hospital of Wenzhou Medical University, Wenzhou, Zhejiang, China

**Keywords:** acute necrotizing encephalopathy of childhood, clinical characteristics, pathogenesis, prognosis, cytokine storm

## Abstract

**Objective:**

Acute Necrotizing Encephalopathy of Childhood (ANEC) is a rare, fulminant neurological disease in children with unknown mechanisms and etiology. This study summarized the clinical characteristics, treatment, and prognosis of ANEC through a retrospective analysis, providing insights into the ANEC early diagnosis and prognosis assessment.

**Methods:**

Clinical data of children diagnosed with ANEC at the Second Affiliated Hospital and Yuying Children’s Hospital of Wenzhou Medical University from July 1, 2020, to June 30, 2023, were retrospectively analyzed.

**Results:**

There were 25 cases, 14 males and 11 females, with a median age of 3 years. Hospital admissions were mainly in the winter (14/25, 56%) and spring (9/25, 36%). All patients presented with varying degrees of fever and altered consciousness, with 92% (23/25) experiencing high body temperatures (>39.1°C) and 88% (22/25) having a Glasgow coma scale (GCS) score of ≤8. Seizures were observed in 88% (22/25) of patients. Laboratory findings indicated 100% B lymphocyte activation (14/14), and 78% (14/18) of patients showed cytokine storm (interleukin (IL)-6, IL-8, IL-10, interferon (IFN)-α). Neuroimaging showed symmetrical thalamus involvement, commonly involving basal ganglia and brainstem regions. Viral infection (23/24, 96%) was the predominant etiological finding, with 42% (10/24) of cases due to SARS-CoV-2 infection and 42% (10/24) to influenza A virus infection. Multi-organ dysfunction occurred in 68% (17/25) of patients, and 52% (13/25) died. Correlation analysis revealed the death group exhibited higher proportion of male, lower GCS scores, higher IL-6 level and a greater likelihood of associated brainstem impairment (*p* < 0.05).

**Conclusion:**

ANEC is more prevalent in the winter and spring, and its etiology may be associated with B lymphocyte activation and cytokine storm following viral infections. Clinical manifestations lack specific features, with fever, consciousness disturbances, and seizures being the main presentations, particularly in cases of high fever and hyperpyrexia. ANEC progresses rapidly and has a high mortality rate. The child’s gender, GCS score, IL-6 levels, and the presence of brainstem involvement can serve as important risk factors for assessing the risk of mortality.

## 1 Introduction

Acute Necrotizing Encephalopathy of Childhood (ANEC), first proposed and named by Mizuguchi et al., is a rare and life-threatening fulminant neurologic disease (1). ANEC primarily occurs in previously healthy children and causes progressive systemic inflammation and brain tissue necrosis. The pathogenesis of ANEC remains unclear; however, current theories suggest that viral infections, overactive immune response following infection, and genetic susceptibility may contribute to ANEC development (2). However, due to its rarity and the complexity of its pathogenesis, the understanding of ANEC is still limited internationally.

The clinical manifestations of ANEC are highly complex and diverse, and the course of the disease is rapid. Typical symptoms include high fever, headache, altered consciousness, seizures, hypomyotonia, and brainstem dysfunction. According to case reports, most ANEC patients have three stages: prodromal infection, acute encephalopathy, and recovery (2, 3). Children commonly have fever and acute respiratory or gastrointestinal symptoms in the prodromal stage, like cough, vomiting, and diarrhea. They then rapidly progress to the encephalopathy stage, displaying progressive deterioration of neurological function, often characterized by altered consciousness, coma, seizure-like episodes, and abnormal muscle tone. According to statistics, the short-term mortality rate of ANEC is as high as 30–50%, and some survivors may have varying degrees of neurological recovery after the acute phase. However, most survivors often suffer from severe neurological sequelae, such as intellectual decline, motor impairments, and cerebral white matter atrophy, requiring long-term rehabilitation (4, 5). The ANEC diagnosis lacks standardized criteria, and the radiological findings of symmetric and multifocal necrotic changes in brain parenchyma are often considered the main diagnostic basis (6). ANEC has no specific treatment; thus, early identification, immunotherapy, and symptomatic support are the main clinical approaches. This study summarized the clinical characteristics, treatment, and prognosis of ANEC through a single-center retrospective analysis, providing insights into the early diagnosis and prognostic evaluation of ANEC.

## 2 Materials and methods

### 2.1 Participants

Clinical data of patients diagnosed with Acute Necrotizing Encephalopathy (ANE) at the Second Affiliated Hospital and Yuying Children’s Hospital of Wenzhou Medical University from July 1, 2020, to June 30, 2023, were retrospectively collected by an electronic medical record management system. Patients aged 29 days to <18 years were enrolled in this study. The diagnostic criteria for ANE were referenced from the literature (1, 6–8), as shown in Table 1. Cases with incomplete data were excluded.

**Table 1:** Diagnostic criteria for acute necrotizing encephalopathy (ANE).

Major criteria	1.Acute encephalopathy following a viral febrile disease. Frequent convulsions and rapid deterioration of consciousness.
	2.CT or MRI evidence of symmetric, multifocal brain lesions. Involvement of the bilateral thalami. Lesions also common in the cerebral periventricular white matter, internal capsule, putamen, upper brain stem tegmentum and cerebellar medulla.
	3.Exclusion of resembling diseases.
Minor criteria	1.Cerebrospinal fluid leucocyte count 8/mm^3^ or less. Elevated or normal protein.
	2.Elevation of serum aminotransferases of variable degrees.
	3.No increase in blood ammonia.

A patient with three major criteria and any two minor criteria would be diagnosed with ANE.

### 2.2 Statistical analysis

Statistical analysis was performed with SPSS version 27.0 (IBM Corporation). Qualitative data were expressed as percentages, and intergroup comparisons were performed using the chi-square or Fisher’s exact test. Quantitative data were presented as the median and quartiles [P50 (P25, P75)]. For normally distributed quantitative data, independent sample *t*-tests were used for intergroup comparisons, while the independent sample Mann–Whitney U test was used for non-normally distributed data. A *p* < 0.05 was considered statistically significant.

## 3 Results

### 3.1 General characteristics

A total of 25 cases were included in the study. The demographic data are shown in Table 2, with 14 males and 11 females. Among them, 17 were urban children, and eight were from scattered rural areas. There was one infant case (29 days to <1 year), nine toddler cases (1 to <3 years), seven preschool cases (3 to <6 years), five school-age cases (6–12 years), and three adolescence cases (12 to <18 years). The median age was 3 years. The median body mass index (BMI) was 15.8, and there were no obese children. Five patients had Guillain-Barré syndrome, central diabetes insipidus, congenital adrenal hyperplasia, epilepsy, and cerebral malformation. Three cases received long-term oral steroid treatment for underlying diseases. All patients had no previous history or family history of necrotizing encephalopathy.

**Table 2:** Demographic data and clinical characteristics.

Characteristic	No. (%)
Gender	
Male	14 (56)
Female	11 (44)
Age, years	3 (2, 6.5)
BMI, kg/m^2^	15.8 (14.3, 19.6)
Place of residence	
Urban	17 (68)
Rural	8 (32)
Season of onset	
Spring (March–May)	9 (36)
Summer (June–August)	1 (4)
Autumn (September–November)	1 (4)
Winter (November–February of the following year)	14 (56)
Clinical presentation	
Fever	25 (100)
High fever (39.1–41.0°C)	15 (60)
Hyperpyrexia (>41.0°C)	8 (32)
Respiratory symptoms	15 (60)
Gastrointestinal symptoms	8 (32)
Disturbance of consciousness	25 (100)
GCS score ≤8	22 (88)
Seizures	22 (88)
Shock	11 (44)
Sluggish or absent pupillary light reflex	20 (80)
Brainstem injury*	17 (68)
Hypomyotonia	13 (52)
Meningeal irritation	10 (40)

Data are shown in n (%) or median (P25, P75). *Patients with brainstem injury referred to those who exhibited both brainstem-related symptoms such as shallow and irregular breathing, as well as abnormal signal changes in the brainstem on neuroimaging. BMI, body mass index; GCS, Glasgow coma scale.

### 3.2 Clinical presentation

The disease onset in the patients was mainly in the winter (14/25, 56%) and spring (9/25, 36%), characterized by acute onset and rapid disease progression. All patients exhibited varying degrees of fever and consciousness impairment. Most patients had high body temperatures, with 60% (15/25) experiencing high fever (39.1–41.0°C) and 32% (8/25) experiencing hyperpyrexia (>41.0°C). Briefly, 88% (22/25) of the patients had a Glasgow coma scale (GCS) score of eight or lower. Seizures were observed in 88% (22/25) of the patients, while shock in 44% (11/25). Respiratory symptoms like cough and sputum production were present in 60% (15/25) of the patients, and gastrointestinal symptoms like diarrhea, abdominal pain, and vomiting were present in 32% (8/25). Upon admission, the main neurological abnormal signs on physical examination were sluggish or absent pupillary light reflex (20/25, 80%), shallow and irregular breathing (17/25, 68%), hypomyotonia (13/25, 52%), and meningeal signs (10/25, 40%), as shown in Table 2.

### 3.3 Auxiliary examinations

**Figure 1:**
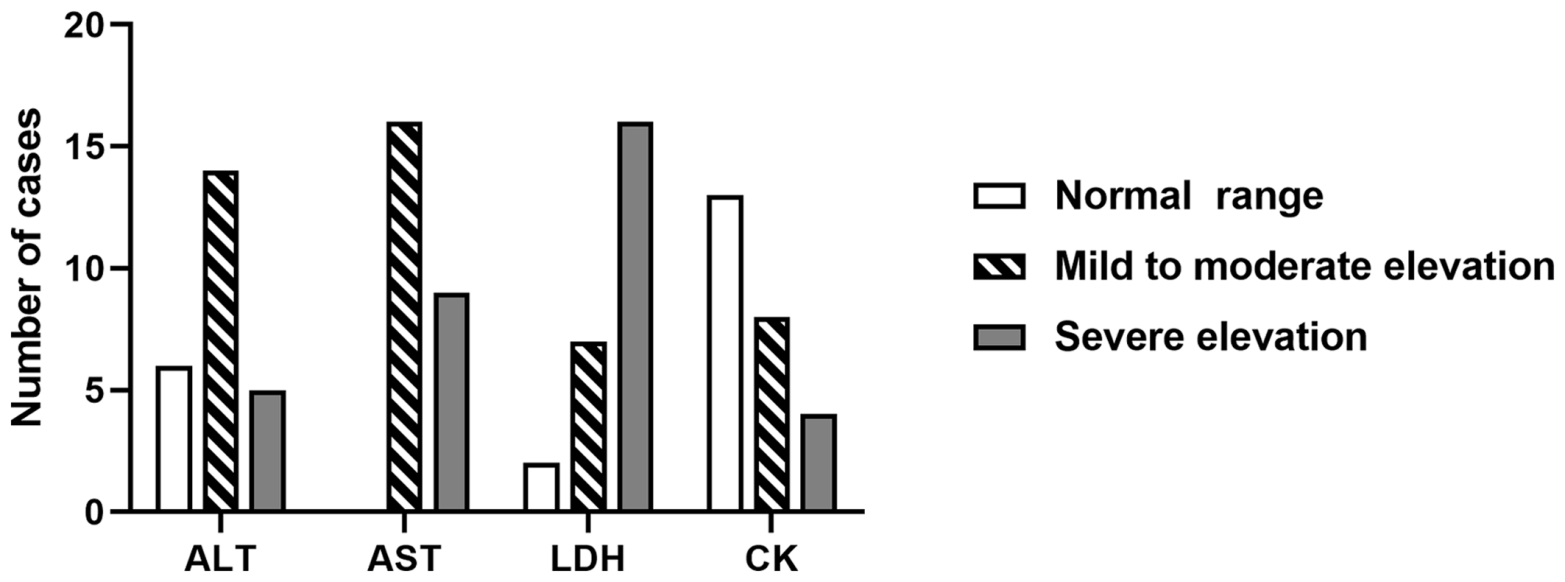
The changes in blood AST, ALT, LDH, and CK levels within 24 h of admission in ANEC patients. AST/ALT: normal range, 5 ~ 40 U/L; mild to moderate elevation, 41 ~ 500 U/L; severe elevation, > 500 U/L. LDH: normal range, 110 ~ 246 U/L; mild to moderate elevation, 246 ~ 500 U/L; severe elevation, > 500 U/L. CK: normal range, 55 ~ 170 U/L, mild and moderate elevation, 170 ~ 500 U/L; severe elevation, > 500 U/L. ALT, alanine aminotransferase; AST, aspartate aminotransferase; LDH, lactate dehydrogenase; CK, creatine kinase isoenzyme.

Comprehensive laboratory examinations, including complete blood count, blood biochemistry, and coagulation function, were conducted for all 25 patients within 24 h of admission, as shown in Table 3. The results revealed that 52% (13/25) of the patients had elevated white blood cell levels, and 16% (4/25) had neutropenia. Thrombocytopenia was occasionally observed. The blood ammonia levels of all patients were normal or slightly above the upper limit, which was clinically insignificant. ANEC patients showed elevated levels of serum enzymes, including alanine aminotransferase (ALT), aspartate aminotransferase (AST), lactate dehydrogenase (LDH), and creatine kinase isoenzyme (CK). ALT and AST were predominantly mildly to moderately elevated, while LDH was predominantly severely elevated, as shown in Figure 1. Cardiac damage was present in 79% (19/24) of the patients, as evidenced by mild elevation of cardiac troponin levels and moderate to severe elevation of B-type natriuretic peptide levels. Metabolic acidosis was observed in 84% (21/25) of the patients, and 46% (11/24) had coagulation dysfunction. Regarding inflammation, C-reactive protein (CRP) levels were mainly mildly elevated, while procalcitonin (PCT) and ferritin levels showed significant elevation. The cellular immune response showed evident activation of CD19+ T-cell (B lymphocytes, 14/14, 100%), and 78% (14/18) of the patients had a cytokine storm [interleukin (IL)-6, IL-8, IL-10, interferon (IFN)-α].

**Table 3:** Laboratory examination results upon admission of 25 ANEC patients.

Laboratory indicators	Median (P25, P75)
White blood cell count (*10^9/L)	12.6 (5.7, 16.2)
Neutrophil count (*10^9/L)	8.7 (3.7, 12.8)
Hemoglobin (g/L)	133 (122.5, 148.5)
Platelet count (*10^9/L)	190 (137.5, 244.5)
Alanine aminotransferase (IU/L)	83 (33, 356.5)
Aspartate aminotransferase (IU/L)	179 (78, 871.5)
Lactate dehydrogenase (IU/L)	751.5 (379.5, 1964.5)
Creatine kinase (IU/L)	146 (95.5, 315.3)
Total bilirubin (μmol/L)	7.8(5.9, 13)
Blood urea nitrogen (mmol/L)	7.8 (6.2, 10.5)
Creatinine (μmol/L)	77(33.5, 118.5)
Albumin (g/L)	41.4 (36.6, 45.8)
Globulin (g/L)	29.6 (26.5, 33.0)
Blood glucose (mmol/L)	5.9 (4.4, 10.7)
Serum calcium (mmol/L)	2.2 (2.0, 2.3)
Serum sodium (mmol/L)	137.8 (135.1, 139.0)
Serum potassium (mmol/L)	4.1 (3.8,4.4)
Troponin (ng/mL)	0.1 (0.03,0.6)
B-type natriuretic peptide (pg/mL)	1880 (765, 2,800)
PH value	7.3 (7.2, 7.4)
Lactic acid (mmol/L)	2.5 (1.4,6.3)
Base excess (mmol/L)	−8.8 (−14.3, −6.3)
PaCO_2_ (mmHg)	40 (29.7, 45.2)
HCO_3_- (mmol/L)	17.1 (12.6, 20.0)
Prothrombin time (PT, s)	19.9 (14.9, 23.5)
International standard ratio (INR)	1.8 (1.2, 2.1)
Activated partial thromboplastin time (APTT, s)	47.2 (39.1, 81.8)
Fibrinogen (FIB, g/L)	2.5 (1.6, 2.9)
D-Dimer (μg/mL)	7.9 (1.7, 20)
C-reactive protein (mg/L)	19.1 (5.5, 34.7)
Procalcitonin (ng/mL)	29.3 (10, 75.7)
Ferritin (ng/mL)	2,000 (1,194, 2,000)
IL-1β (pg/mL)	2.5 (2.5, 110.1)
IL-2 (pg/mL)	2.5 (2.5, 4.1)
IL-4 (pg/mL)	2.5 (2.5, 2.7)
IL-5 (pg/mL)	2.5 (2.5, 2.5)
IL-6 (pg/mL)	91.3 (9.9, 1162.4)
IL-8 (pg/mL)	126.4 (13.9, 12098.3)
IL-10 (pg/mL)	108.8 (14.3, 349.0)
IL-12P70 (pg/mL)	2.5 (2.5, 2.5)
IL-17A (pg/mL)	10 (10, 11.4)
IFN-γ (pg/mL)	2.5 (2.5, 4.7)
TNF-α (pg/mL)	3.3 (2.5, 4.7)
IFN-α (pg/mL)	30.1 (5.2, 183.4)
CD3+ T-cell percentage (%)	48.1 (41.9, 56.1)
CD4+ T-cell percentage (%)	19.9 (16.4, 30.4)
CD8+ T-cell percentage (%)	19.2 (16.2, 22.2)
CD4/CD8 ratio	1.0 (0.9, 1.7)
CD16 + 56+ T-cell percentage (%)	10.3 (6.6, 17.4)
CD19+ T-cell percentage (%)	36.9 (29.7, 44.6)
CSF protein (g/L)	0.5 (0.2, 1.7)
CSF- white blood cell count (*10^6/L)	1 (0, 2.3)
CSF- chloride (mmol/L)	126.3 (119.8, 128.9)
CSF- glucose (mmol/L)	4.9 (3.6, 6.6)

IL, interleukin; IFN, interferon; TNF, tumor necrosis factor; CSF, cerebrospinal fluid.

**Figure 2:**
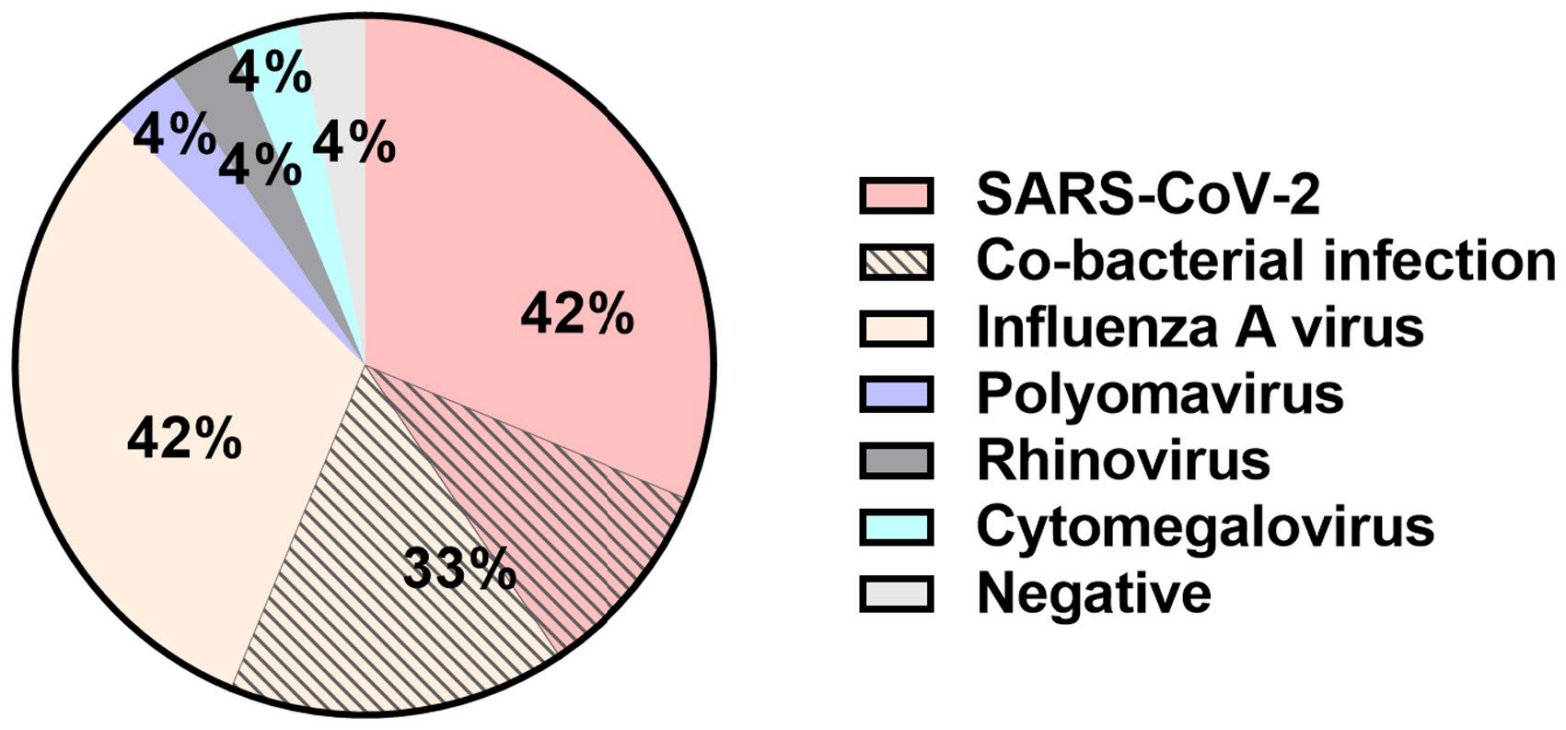
Distribution of pathogen in 24 ANEC patients.

Respiratory viral antigen testing, respiratory viral nucleic acid testing, sputum bacterial culture, blood bacterial culture, and other pathogen tests were conducted on 24 patients, with an overall positivity rate of 96% (23/24), as shown in Figure 2. Additionally, one patient was admitted in critical condition but succumbed to multiple organ dysfunction syndrome (MODS) shortly after admission despite rescue efforts. The pathogen analysis revealed that respiratory viral infections (22/24, 92%) were the predominant findings in ANEC patients. Among them, 42% (10/24) of the patients were infected with SARS-CoV-2, 42% (10/24) with influenza A virus, 21% (5/24) had concomitant positive sputum bacterial cultures, and 13% (3/24) had concomitant positive blood bacterial cultures. In patients with concomitant bacterial infections, 88% (7/8) of the infecting strains were Gram-positive, with Staphylococcus species accounting for six cases.

**Figure 3:**
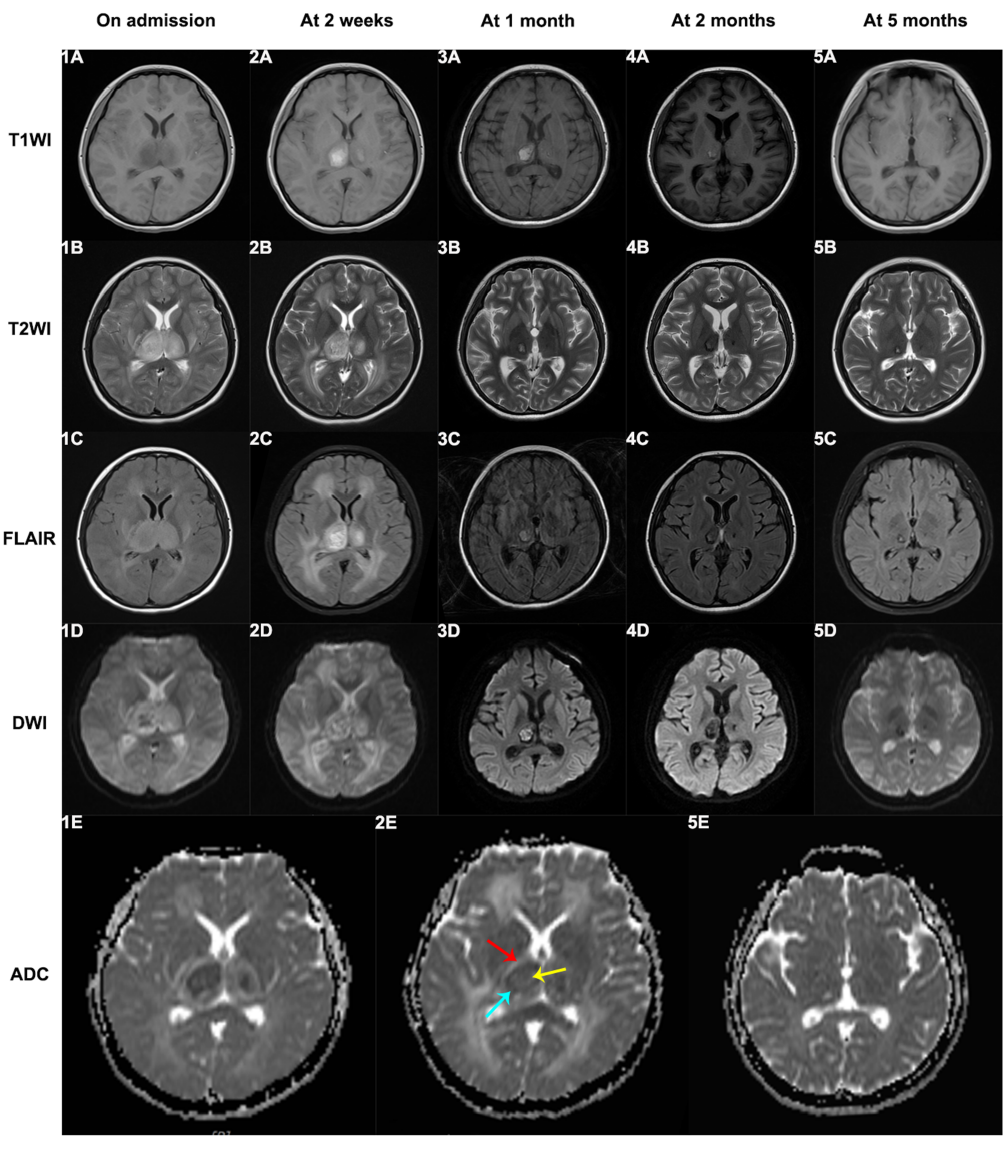
The MRI changes of thalamic lesions throughout the disease in a 13-year-old female ANEC patient. Initially, the lesion area appeared isointense or slightly hypointense on T1WI, slightly hyperintense on T2WI and FLAIR sequence, and heterogeneously hyperintense on DWI. The ADC map displayed a typical concentric ring-shaped abnormal signal in the thalamus, with the outer layer showing a high signal indicating vascular edema (indicated by red arrow), the middle layer showing a low signal indicating cytotoxic edema (indicated by blue arrow), and the inner layer showing slight elevation in ADC signal indicating hemorrhage and necrosis (indicated by yellow arrow). T1WI, T1-weighted imaging; T2WI, T2-weighted imaging; FLAIR, fluid-attenuated inversion recovery; DWI, diffusion-weighted imaging; ADC, apparent diffusion coefficient.

Sixteen patients underwent cerebrospinal fluid (CSF) examinations after admission. Most ANEC patients showed elevated protein content in the CSF, positive Pandy test, normal white blood cell count, normal chloride levels, and no decrease in glucose levels. Pathogen detection in the CSF was negative, indicating no direct evidence of pathogen invasion. Neuroimaging findings in ANEC patients exhibited symmetric thalamus involvement, involving other regions like the basal ganglia and brainstem. Figure 3 depicts the MRI changes of thalamic lesions throughout the disease in a 13-year-old female ANEC patient. Initially, the lesion area appeared isointense or slightly hypointense on T1-weighted imaging (T1WI), slightly hyperintense on T2-weighted imaging (T2WI) and fluid-attenuated inversion recovery (FLAIR) sequence, and heterogeneously hyperintense on diffusion-weighted imaging (DWI). The apparent diffusion coefficient (ADC) map displayed a typical concentric ring-shaped abnormal signal in the thalamus, with the outer layer showing a high signal indicating vascular edema, the middle layer showing a low signal indicating cytotoxic edema, and the inner layer showing slight elevation in ADC signal indicating hemorrhage and necrosis (9, 10). Most patients reached the peak of brain lesions around the second week of the disease course, with MRI indicating worsening cerebral hemorrhage and necrosis. After the acute phase, patients entered the recovery phase at 1 month, with older lesions being absorbed and MRI data suggesting old hemorrhagic lesions. Thalamic lesions showed significant regression at 2 and 5 months of follow-up, with MRI indicating iron deposition. Electroencephalogram (EEG) changes in ANEC patients were mainly characterized by background rhythm slowing, and some patients exhibited epileptic discharges. Bedside EEG monitoring showed bilateral cerebral hemisphere resting potentials with no response to stimulation in 5 cases.

### 3.4 Treatment

Antipyretics, mannitol for intracranial pressure reduction, seizure control, and maintenance of a stable internal environment were given to all patients after admission. 56% (14/25) of the patients received antiviral therapy, with all influenza A patients receiving oseltamivir treatment. About 88% (22/25) of the patients received empirical antibiotic treatment within the first 3 days of the disease course, with cefoperazone-sulbactam (12/22, 55%) and ceftriaxone (7/22, 32%) being the main choices. About 96% (24/25) of patients initiated immunotherapy within 24 h of hospital admission. About 76% (19/25) of the patients received steroid pulse therapy, and 48% (12/25) received high-dose intravenous immunoglobulin therapy. Moreover, 64% (16/25) underwent endotracheal intubation, 32% (8/25) underwent plasma exchange, 32% (8/25) received hypothermia treatment, and 16% (4/25) received continuous renal replacement therapy (CRRT), as shown in Table 4.

**Table 4:** Treatment and prognosis of 25 ANEC patients.

Treatment	No. (%)
Methylprednisolone	23 (92)
2–3 mg·kg^−1^·day^−1^	4 (16)
10–20 mg·kg^−1^·day^−1^	19 (76)
Intravenous immunoglobulin	23 (92)
400 mg·kg^−1^·day^−1^ *5 days	11 (44)
1,000 mg·kg^−1^·day^−1^ *2 days	12 (48)
Tracheal intubation	16 (64)
Plasma exchange	8 (32)
Mild hypothermia therapy	8 (32)
CRRT	4 (16)
Antiviral therapy	14 (56)
Oseltamivir	10 (71)
Baloxavir	1 (7)
Ganciclovir	3 (21)
Antibiotic therapy	22 (88)
MODS	17 (76)
Clinical outcomes	
Death	13 (52)
Survival	12 (48)
Total length of hospital stay for surviving children, days	17 (9.3, 29.2)

Data are shown in n (%) or median (P25, P75). CRRT, continuous renal replacement therapy; MODS, multiple organ dysfunction syndrome.

### 3.5 Prognosis

MODS developed in 76% (17/25) of patients, and 52% (13/25) died. Most deaths occurred during the acute encephalopathy phase, with a median hospital stay of 2 days for deceased patients and 17 days for survivors. It is worth noting that among the 13 children included in the group that unfortunately died, 5 succumbed to their condition while still admitted in the hospital, while the remaining 8 were critically ill children. Given the grim outlook, their families made the difficult decision to forgo discharge and opted for end-of-life care outside the hospital setting. Therefore, these patients were also classified into the death group. A comparison of the clinical characteristics and laboratory findings between the ANEC patients in the death group and the improvement group is shown in Tables 5, 6. There were no statistically significant differences in age, BMI, season of onset, peak body temperature, seizure proportion, SARS-CoV-2/ influenza A virus infection proportion, or treatment outcomes (*p* > 0.05). However, the proportion of males was significantly higher in the death group (10/13, 76.9%) compared to the improvement group (4/12, 33.3%), and the death group had lower GCS scores, higher shock rates, myocardial damage, and MODS, and a higher tracheal intubation demand (*p* < 0.05). Regarding physical signs, the death group was more likely to exhibit sluggish or absent pupillary light reflex, hypomyotonia, and brainstem impairment, with statistically significant differences (*p* < 0.05). Laboratory results showed greater hemoglobin, creatinine, and IL-6 levels in the death group of ANEC patients. Additionally, they showed more severe acidosis, coagulation failure, and decreased blood calcium levels, with all differences being statistically significant (*p* < 0.05).

**Table 5:** Comparison of clinical characteristics between the death and improvement groups in ANEC patients.

Characteristic	Death group (*n* = 13)	Improvement group (*n* = 12)	*p*-value
**Gender**			**0.028**
**Male**	**10 (76.9)**	**4 (33.3)**	
**Female**	**3 (23.1)**	**8 (66.7)**	
Age, years	4 (2, 8)	2.7 (1.6, 6.3)	0.512
BMI, kg/m^2^	15.8 (14.1, 18.7)	16.8 (14.7, 20.5)	0.311
Season of onset			0.557
Spring (March–May)	5 (38.5)	4 (33.3)	
Summer (June–August)	1 (7.7)	0 (0)	
Autumn (September–November)	0 (0)	1 (8.3)	
Winter (November–February of the following year)	7 (53.8)	7 (58.3)	
Clinical presentation			
Fever	13 (100)	12 (100)	–
High fever (39.1–41.0°C)	8 (61.5)	7 (58.3)	0.87
Hyperpyrexia (>41.0°C)	5 (38.5)	3 (25)	0.471
Respiratory symptoms	7 (53.8)	8 (66.7)	0.513
Disturbance of consciousness	13 (100)	12 (100)	–
**GCS scores**	**2 (2, 2.5)**	**6.5 (3.3, 8.8)**	**<0.001**
Seizures	11 (84.6)	11 (91.7)	0.588
Shock	9 (69.2)	2 (16.7)	**0.008**
Sluggish or absent pupillary light reflex	13 (100)	7 (58.3)	**0.009**
Brainstem injury*	12 (92.3)	5 (41.7)	**0.007**
Hypomyotonia	10 (76.9)	3 (25)	**0.009**
Meningeal irritation	3 (23.1)	7 (58.3)	0.072
**Myocardial damage**	**13 (100)**	**6 (54.5)**	**0.006**
**MODS**	**12 (92.3)**	**5 (41.7)**	**0.007**
**Total length of hospital stay, days**	**2 (2, 2.5)**	**17 (9.3, 29.3)**	**<0.001**
Viral infection			
SARS-CoV-2	6 (54.5)	4 (50)	0.845
Influenza A virus	5 (62.5)	5 (71.4)	0.714
Treatment			
Methylprednisolone	11 (84.6)	12 (100)	0.157
Dose schedule			0.924
2–3 mg·kg^−1^·day^−1^	2 (18.2)	2 (16.7)	
10–20 mg·kg^−1^·day^−1^	9 (81.8)	10 (83.3)	
Intravenous immunoglobulin	12 (92.3)	11 (91.7)	0.953
Dose schedule			0.292
400 mg·kg^−1^·day^−1^*5 days	7 (58.3)	4 (36.4)	
1,000 mg·kg^−1^·day^−1^*2 days	5 (41.7)	7 (63.6)	
**Tracheal intubation**	**13 (100)**	**3 (25)**	**<0.001**
Plasma exchange	4 (30.8)	4 (33.3)	0.891
Mild hypothermia therapy	5 (38.5)	3 (25)	0.471
CRRT	2 (15.4)	2 (16.7)	0.93
Antiviral therapy	6 (46.2)	8 (66.7)	0.302
Antibiotic therapy	11 (84.6)	11 (91.7)	0.588

Data are shown in median (P25, P75) or n (%). *Patients with brainstem injury referred to those who exhibited both brainstem-related symptoms such as shallow and irregular breathing, as well as abnormal signal changes in the brainstem on neuroimaging. BMI, body mass index; GCS, Glasgow coma scale. Bold values indicate the items with a statistically significant difference (*p* < 0.05).

**Table 6:** Comparison of laboratory findings between the death and improvement groups in ANEC patients.

Laboratory indicators	Death group (*n* = 13)	Improvement group (*n* = 12)	*P*-value
White blood cell count (*10^9/L)	12.6 (5.7, 16.4)	12.8 (3.9, 16.2)	0.913
Neutrophil count (*10^9/L)	8.6 (3.7, 15.3)	9.2 (2.7, 12.0)	0.947
**Hemoglobin (g/L)**	**141 (128, 158)**	**128.5 (109, 135.5)**	**0.019**
Platelet count (*10^9/L)	158 (116, 223.5)	223 (144.5, 284)	0.063
Alanine aminotransferase (IU/L)	101 (46, 465)	55.5 (29.8, 235.8)	0.369
Aspartate aminotransferase (IU/L)	426 (115, 1, 105)	93.5 (61.8, 802.5)	0.174
Lactate dehydrogenase (IU/L)	1, 000 (616, 2, 398)	457 (313, 1, 447)	0.06
Creatine kinase (IU/L)	150 (105.5, 404.5)	142 (90, 318)	0.772
Total bilirubin (μmol/L)	9.7 (6.9, 13.6)	6.9 (5.3, 9.4)	0.276
Blood urea nitrogen (mmol/L)	7.7 (6.2, 11.5)	8.0 (5.4, 9.7)	0.813
**Creatinine (μmol/L)**	**107 (62, 142.5)**	**39.1 (19.1, 98.5)**	**0.011**
Albumin (g/L)	40 (36.0, 43.1)	45 (38.8, 46.9)	0.07
Globulin (g/L)	29.6 (26.2, 33.2)	29.1 (26.5, 32.9)	0.487
Blood glucose (mmol/L)	6.6 (3.2, 11.4)	5.8 (4.5, 10.8)	0.802
**Serum calcium (mmol/L)**	**2.1 (1.9, 2.2)**	**2.28 (2.1, 2.4)**	**0.013**
Serum sodium (mmol/L)	138.5 (135.1, 141.5)	137.3 (135, 138.9)	0.839
Serum potassium (mmol/L)	4.0 (3.7, 4.3)	4.1 (3.8, 4.5)	0.597
**Troponin (ng/mL)**	**0.5 (0.06, 0.8)**	**0.04 (0, 0.2)**	**0.029**
B-type natriuretic peptide (pg/mL)	1990 (1330.5, 2, 790)	1, 006 (160.8, 4, 505)	0.172
PH value	7.3 (7.1, 7.3)	7.3 (7.2, 7.4)	0.084
**Lactic acid (mmol/L)**	**5.1 (2.1, 9.2)**	**1.6 (1.2, 3.4)**	**0.015**
**Base excess (mmol/L)**	**−11.1 (−15.7, -8.6)**	**−6.6 (−9.5, -2)**	**0.009**
PaCO_2_ (mmHg)	39 (26.8, 49.9)	40 (28.7, 45.6)	0.828
**HCO**_3_**-** (mmol/L)	**14.5 (12.5, 17.3)**	**19.8 (16.9, 23.0)**	**0.009**
**Prothrombin time (PT, s)**	**22.2 (18.1, 27.8)**	**15.4 (13.7, 20.8)**	**0.016**
**International standard ratio (INR)**	**2 (1.6, 2.8)**	**1.2 (1.1, 1.9)**	**0.019**
**Activated partial thromboplastin time (APTT, s)**	**80.2 (41.8, 113.4)**	**41.7 (38.3, 53)**	**0.034**
Fibrinogen (FIB, g/L)	2.4 (1.5, 2.8)	2.6 (2.1, 3.0)	0.192
**D-Dimer (μg/mL)**	**20 (3.0, 20)**	**2.8 (0.8, 12)**	**0.038**
C-reactive protein (mg/L)	13.23 (4.4, 28.9)	19.295 (4.5, 52.7)	0.664
Procalcitonin (ng/mL)	29.77 (13.2, 87.8)	15.87 (0.2, 57.9)	0.236
Ferritin (ng/mL)	2000 (1202.8, 47093.8)	1, 500 (1, 194, 1971.5)	0.463
IL−1β (pg/mL)	5 (2.5, 167.5)	2.5 (2.5, 18.3)	0.174
IL-2 (pg/mL)	2.5 (2.5, 4.3)	2.5 (2.5, 4.0)	0.88
IL-4 (pg/mL)	2.5 (2.5, 2.6)	2.5 (2.5, 3)	0.752
IL-5 (pg/mL)	2.5 (2.5, 2.5)	2.5 (2.5, 2.5)	0.398
**IL-6 (pg/mL)**	**538.1 (69.0, 8688.5)**	**10.99 (3.7, 120.3)**	**0.015**
IL-8 (pg/mL)	287.8 (56.6, 18444.6)	23.76 (8.3, 1130.1)	0.088
IL-10 (pg/mL)	177.7 (59.7, 684.6)	22.7 (8.8, 218.2)	0.145
IL-12P70 (pg/mL)	2.5 (2.5, 2.5)	2.5 (2.5, 2.5)	1
IL-17A (pg/mL)	10 (10, 10)	10 (10, 15.4)	0.456
IFN-γ (pg/mL)	3.4 (2.5, 8.5)	2.5 (2.5, 3.8)	0.196
TNF-α (pg/mL)	4.4 (2.5, 11.4)	2.9 (2.5, 3.6)	0.133
IFN-α (pg/mL)	16.5 (2.5, 294.5)	42.2 (10.2, 176.8)	0.871
CD3+ T-cell percentage (%)	46.7 (44.1, 57.7)	51.6 (36.3, 56.1)	0.836
CD4+ T-cell percentage (%)	18.6 (15.3, 28.4)	24.8 (17.5, 32.8)	0.434
CD8+ T-cell percentage (%)	21.5 (16.9, 25.8)	17.8 (13.8, 20.4)	0.110
CD4/CD8 ratio	1.0 (0.7, 1.5)	1.4 (1.0, 2.1)	0.124
CD16 + 56+ T-cell percentage (%)	9.5 (7.3, 11.8)	13.7 (5.7, 21.3)	0.519
CD19+ T-cell percentage (%)	41.2 (30.0, 44.8)	35.7 (29.6, 43.4)	0.439
CSF protein (g/L)	2.4 (0.3, 2.7)	0.4 (0.2, 0.7)	0.139
CSF- white blood cell count (*10^6/L)	0 (0, 0)	2 (0.5, 2.5)	0.126
CSF- chloride (mmol/L)	126.4 (126.1, 133.0)	123 (119, 128.4)	0.312
CSF- glucose (mmol/L)	5.3 (4.3, 6.3)	4.9 (3.5, 6.7)	0.814

Data are shown in median (P25, P75) or n (%). IL, interleukin; IFN, interferon; TNF, tumor necrosis factor; CSF, cerebrospinal fluid. Bold values indicate the items with a statistically significant difference (*p* < 0.05).

## 4 Discussion

Acute necrotizing encephalopathy of childhood (ANEC) is a rare central nervous system disease that results in severe neurological damage or death. This study retrospectively assessed ANEC patients’ clinical data from a large comprehensive hospital in China over 3 years. ANEC predominantly affects children in the toddler and preschool age group (1–6 years), with a slightly higher proportion of males, who are also at a greater risk of progressing to severe illness or death. The onset of ANEC exhibits a significant seasonal pattern, with a higher incidence in the winter and spring. However, the clinical manifestations in affected children are not specific, with hyperpyrexia, severe coma, and seizures being the main presenting features. In 68% (17/25) of the patients, there were signs of brainstem impairment, and cases with concomitant brainstem impairment had a higher mortality rate, consistent with the findings of Okumura et al. (11).

The etiology of ANEC remains unclear; however, current theoretical hypotheses suggest a close association with infection, immune abnormalities, and genetic factors. Numerous studies have indicated that infection is a common triggering factor for ANEC. Various pathogens, like influenza viruses, human herpesviruses, and enteroviruses, have been found in ANEC patients and are associated with the onset of ANEC (2). Infection can activate the body’s immune system, leading to an excessive inflammatory response and neuronal damage. However, no evidence suggests direct invasion of the brain by pathogens. Our findings are consistent with previous research, as all our 25 ANEC patients had a history of preceding infection, with predominant respiratory viral infections. In our study, influenza A virus and SARS-CoV-2 were the main pathogens associated with disease onset. The 16 patients who had CSF examinations showed no direct pathogen infection.

Moreover, genetic factors are also important contributing factors to ANEC. Most cases of ANE are sporadic and non-recurrent (isolated ANE). Still, more reports have indicated familial aggregation in some cases, and further genetic testing has identified pathogenic gene mutations in affected families linked to cellular mitochondrial energy metabolism and immune dysregulation. For example, in 2009, Neilson et al. first reported that missense mutations in the RAN binding protein 2 (RANBP2) gene can lead to hereditary (familial) ANE, called ANE1. This gene encodes a multifunctional protein crucial in nuclear-cytoplasmic transport in brain tissues, participating in mitochondrial intracellular transport, energy production, lipid peroxidation, and maintaining the integrity of the blood–brain barrier (12). In 2016, researchers from Japan (13) and in 2019, researchers from Korea (6) proposed that polymorphic human leukocyte antigen (HLA) genotypes may be associated with a predisposition to ANE. HLA genotypes consist of HLA class I (HLA-A, -B, and -C) and class II (HLA-DR, -DQ, and -DP) molecules, which are vital in regulating acquired/innate immune responses and self-recognition/non-self-recognition. Additionally, studies have also reported an association between mutations in the carnitine palmitoyltransferase 2 (CPT2) gene and ANE. CPT2 is located on the mitochondrial inner membrane and is a key enzyme involved in lipid metabolism (14). In our study, the majority of ANEC cases were isolated ANE, and due to the retrospective design limitations, we have not yet completed ANE-related genetic testing in the patients.

**Figure 4:**
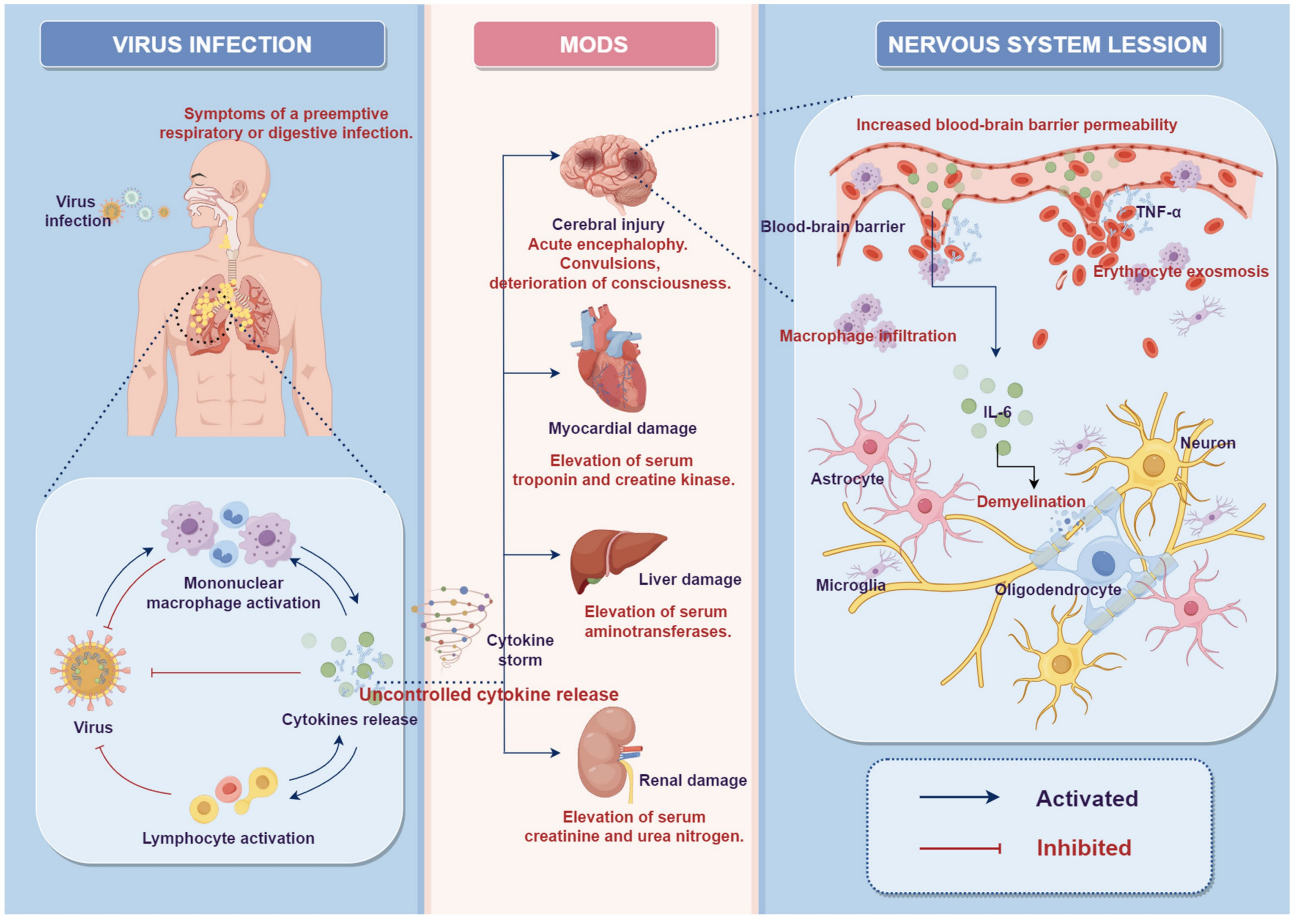
Potential role of “cytokine storm” in the pathophysiology of ANEC (by Figdraw 2.0). After the viral invasion of the body, the local activation of the mononuclear macrophage system leads to the secretion of cytokines and further activation of lymphocytes, playing a role in antiviral defense. Under certain conditions, this response may become dysregulated, leading to excessive release of inflammatory cytokines, resulting in a cytokine storm, which causes systemic multi-organ damage and dysfunction. In the nervous system, excessive inflammatory cytokines can increase blood–brain barrier permeability, leading to intracranial vascular lesions, erythrocyte exosmosis, and infiltration of macrophages. This can lead to mild activation and proliferation of astrocytes and microglia. It can also cause damage to oligodendrocytes and axons, promote demyelination, and result in neuronal atrophy and necrosis. MODS, multiple organ dysfunction syndrome.

In the histopathology of autopsies on previously reported ANEC cases, brain tissue mainly exhibited diffuse edema, focal hemorrhagic necrosis, and cerebrovascular abnormalities (9, 15–17). Consistent with the radiological findings, focal hemorrhagic necrosis in brain tissue was characterized by punctate hemorrhage and necrosis in the thalamus. The cerebrovascular abnormalities predominantly manifested as vascular exudative changes, characterized by dilated perivascular spaces with fibrous exudates often associated with vacuolar artifacts (15). Histologically, neuronal atrophy, necrosis and demyelination were observed. There was a significant infiltration of macrophages around blood vessels, and mildly activated proliferation of astrocytes and microglia was evident (9, 16, 17). These cases’ radiological and autopsy findings strongly suggested blood–brain barrier (BBB) disruption. Furthermore, the degree of disruption gradually increased with depth from the brain surface. Mizuguchi et al. observed that all caliber vessels exhibited severe extravasation of red blood cells in the central part of the lesions, i.e., perivascular hemorrhage (9).Some clinical research has detected cytokine storms in ANEC patients’ blood and CSF in recent years. These cytokines include IL-6, tumor necrosis factor (TNF)-α, and IL-1β (18–21). Researchers have also found a positive correlation between elevated IL-6 levels and ANEC severity and degree of neurological damage in ANEC patients (22). Therefore, the theory of cytokine storm has been proposed, suggesting that it may be vital in the ANEC pathogenesis. Cytokines are a type of secreted protein that can regulate immune responses, cell proliferation, and cell death, among other functions. Under normal circumstances, the cytokines in the body maintain a balanced state. However, when the body is subjected to infection, injury, or other stimuli, the immune system releases many cytokines in response. However, in certain situations, this response may become dysregulated, leading to excessive release of inflammatory factors and the occurrence of a cytokine storm. This storm hits the body’s vasculature, increasing vascular permeability and causing secondary damage and dysfunction in multiple organs throughout the body (23, 24). The theoretical hypothesis suggests that the accumulation of cytokines following viral infection and subsequent disruption of the BBB is a major pathogenic mechanism of ANEC (25, 26), as depicted in Figure 4. IL-6 is believed to have neurotoxic effects at high concentrations, promoting demyelination of neurons and causing damage to oligodendrocytes and axons (27). TNF-α can impair the endothelium of blood vessels in the central nervous system, leading to increased BBB permeability (28). Under normal circumstances, BBB restricts harmful substances from entering the central nervous system. However, in ANEC patients, the cytokine storm increases vascular permeability of the brain blood vessels, causing vascular lesions characterized by initial fibrinous exudation, progressing to extravasation of red blood cells, resulting in cerebral edema, hemorrhage, and subsequent neuronal death (9, 15). Furthermore, the cytokine storm can induce activation and proliferation of macrophages. The damage to the BBB allows various harmful substances and inflammatory factors to enter the brain tissue. Over-inflammation damages neuron bio membranes, releasing toxic chemicals and causing more neuronal death (16, 17, 29). Consistent with previous research findings, we found a significant increase in IL-6, 8, 10, and IFN-α, in the serum of ANEC patients. Additionally, we observed that the death group had higher IL-6 levels than the group of patients who experienced improvement, with statistically significant differences. In our cases, 76% (17/25) of the patients eventually developed MODS, and the death group had an even higher incidence of MODS, reaching 92.3% (12/13). Cytokine storm is an acute systemic inflammatory syndrome that can lead to multiple organ failure, and the involvement of multiple target organs in MODS further confirms the existence of cytokine storm. We examined lymphocyte activation levels in the body further. Our results revealed that ANEC patients, in addition to producing cytokine storms, exhibited significant B lymphocyte activation. The immunological response relies on B lymphocyte-cytokine interaction. Cytokines act as signaling molecules that regulate the activation, differentiation, and function of B lymphocytes. Activated B lymphocytes produce many cytokines and participate in immune regulation, inflammatory responses, and hematopoiesis. However, it should be noted that the pathological process and molecular mechanisms of ANEC described above are current theoretical hypotheses. Intervention in the cytokine signaling pathway and immune system activation may prevent and treat ANEC, but further research is still needed to validate and refine these hypotheses. This includes in-depth analysis of patients’ genetic, immunological, and cellular characteristics, as well as the establishment of animal models and further exploration of biomolecular research.

There is still a lack of specific treatment methods and standardized treatment protocols for ANEC in clinical practice. The treatment principles mainly focus on immune modulation and symptomatic support. Immune therapy involves first-line treatments like steroids, and intravenous immunoglobulins and second-line treatments like Tocilizumab (30). However, the specific efficacy of immune therapy remains controversial. It is generally believed that steroids suppress the inflammatory process, including increased cytokine levels and vascular hyperpermeability. If administered at an appropriate time, steroids may improve the prognosis of ANEC. A study by Li et al. showed that high-dose methylprednisolone pulse therapy (initial dose ≥20 mg·kg-1·day-1) can reduce the mortality rate of ANEC children at discharge (4). A Japanese multicenter study indicated early steroid use is effective only in ANEC patients without brainstem lesions. In contrast, with brainstem lesions, any treatment seems to have no significant impact, and brainstem lesions may be a poor prognostic indicator (11). Delamarre et al. reported that early combined use of high-dose steroids and intravenous immunoglobulin therapy has a significant therapeutic effect in preventing the progression of ANE (31). However, Okumura et al. suggested that the therapeutic effect of intravenous immunoglobulin is limited (11). As indicated, elevated IL-6 may cause ANEC. Tocilizumab, a monoclonal antibody targeting the IL-6 receptor, may treat ANEC. In recent years, several case reports have confirmed that adding Tocilizumab as an “adjunct” immunotherapy to steroids and immunoglobulin therapy has shown promising therapeutic effects in the medium to long-term prognosis of ANEC (22, 32, 33). However, Tocilizumab is limited in children due to its various adverse reactions, including liver dysfunction, allergic reactions, and rare gastrointestinal perforation (34). Additionally, opinions suggest that plasma exchange may be beneficial in reducing mortality rates (35). CRRT can reduce inflammatory cytokines, thereby improving brain function (36). Hypothermia can lower brain metabolism and cerebral blood flow while reducing pro-inflammatory factors like IL-6 and TNF-α (37). It has shown good results in ANEC cases (38), but further research is needed to confirm these findings. In this study, almost all patients (24/25, 96%) initiated immune therapy within 24 h of admission, including steroid pulse therapy, high-dose intravenous immunoglobulin therapy, plasma exchange, hypothermia treatment, and CRRT. However, a retrospective analysis of our cases found no statistically significant difference in comparing these treatment regimens between the death and improvement groups (*p* > 0.05). This could be attributed to the small sample size and potential bias. Additionally, our center is a regional pediatric critical care transport center with a relatively higher proportion of critically ill patients. Brainstem injury accounted for 68% (17/25) of the cases, and as mentioned by Okumura et al., the therapeutic efficacy of the medication is limited in severe cases (11). Furthermore, in our cases, all influenza-related ANEC patients received oseltamivir treatment. However, the standard dose of oseltamivir (2 mg·kg^−1^·day^−1^) did not significantly impact the outcome of ANEC patients. Alsolami and Shiley reported a case of an adult ANE patient who showed significant improvement in psychiatric symptoms after high-dose oseltamivir treatment (150 mg twice daily). They proposed the suggestion of using high-dose oseltamivir for at least 14 days in influenza-related ANE patients (39). The oseltamivir treatment strategy and dose for influenza-associated ANEC children may need more study.

However, the use of immune modulation therapy in ANEC children remains a topic of ongoing research and discussion, and the optimal dosage and regimen of these therapies have yet to be determined. The potential benefits of immune modulation therapy should be balanced with the associated risks. These therapies can alleviate inflammation and tissue damage but hinder normal immune function and increase infection risk. Therefore, when using immunosuppressive drugs to manage ANEC, close monitoring of immune function and adverse reactions is necessary. Infection prevention and control measures should be implemented, and strict control of drug dosage and regimen should be maintained to ensure the safety and effectiveness of treatment. Since ANEC rapidly worsens, early discovery, precise diagnosis, and appropriate intervention improve prognosis and survival for ANEC children.

In summary, ANEC is a rare pediatric critical illness with unclear pathogenesis and a lack of effective and standardized treatment methods. This study analyzed the clinical data of ANEC patients from a single center over 3 years and, based on the results, reviewed and summarized the current research progress on the pathogenesis and treatment of ANEC. The cytokine storm hypothesis suggests that the uncontrolled release of pro-inflammatory cytokines following viral infection, particularly IL-6, is crucial in ANEC pathogenesis. This phenomenon leads to disruption of the BBB, endothelial damage, and neuronal death, ultimately manifesting as the severe cerebral disease characteristic of ANEC. Understanding and targeting the cytokine storm phenomenon may provide potential therapeutic approaches for managing or preventing the progression of this severe neurologic disorder. However, further research is needed for a deeper understanding of the association between the cytokine storm and ANEC to find more effective treatment strategies.

This study is limited by its retrospective approach and small patient population. Large-scale randomized controlled trials with greater sample sizes are needed to prove immunotherapy efficacy. However, the extremely low incidence rate of ANEC makes it challenging to conduct randomized controlled trials. To gain a more comprehensive understanding of ANEC, future research can strengthen multi-center collaborations, increase the sample size, and conduct long-term follow-up observations. Furthermore, attention should be paid to in-depth analysis of the genetic, immunological, and cytological aspects of patient samples, the establishment of animal models, and further exploration of biomolecular research.

## Data availability statement

The raw data supporting the conclusions of this article will be made available by the authors, without undue reservation.

## Ethics statement

The studies involving humans were approved by the Medical Ethics Committee of the Second Affiliated Hospital and Yuying Children’s Hospital of Wenzhou Medical University. The studies were conducted in accordance with the local legislation and institutional requirements. Written informed consent from the patients/participants or patients/participants legal guardian/next of kin was not required to participate in this study in accordance with the national legislation and the institutional requirements.

## Author contributions

YF: Writing – original draft, Writing – review & editing. QG: Writing – original draft, Writing – review & editing. WJ: Writing – original draft. JL: Writing – original draft. HY: Writing – original draft. ZL: Writing – review & editing. GP: Writing – review & editing. WL: Writing – review & editing, Conceptualization, Investigation, Methodology, Supervision.
